# Simple, sensitive and quantitative bioluminescence assay for determination of malaria pre-patent period

**DOI:** 10.1186/1475-2875-13-15

**Published:** 2014-01-08

**Authors:** Vanessa Zuzarte-Luis, Joana Sales-Dias, Maria M Mota

**Affiliations:** 1 Instituto de Medicina Molecular, Faculdade de Medicina Universidade de Lisboa, Lisboa 1649-028, Portugal

## Abstract

**Background:**

The first phase of malaria infection occurs in the liver and is clinically silent. Inside hepatocytes each *Plasmodium* sporozoite replicate into thousands of erythrocyte-infectious merozoites that when released into the blood stream result in clinical symptoms of the disease. The time between sporozoite inoculation and the appearance of parasites in the blood is defined as the pre-patent period, which is classically analysed by time-consuming and labor-intensive techniques, such as microscopy and PCR.

**Methods:**

Luciferase-expressing *Plasmodium berghei* parasites were used to measure pre-patent period of malaria infection in rodents using a bioluminescence assay that requires only one microliter of blood collected from the tail-vein. The accuracy and sensitivity of this new method was compared with conventional microscopy and PCR based techniques, and its capacity to measure the impact of anti-malarial interventions against the liver evaluated.

**Results:**

The described method is very sensitive allowing the detection of parasites during the first cycles of blood stage replication. It accurately translates differences in liver load due to inoculation of different sporozoite doses as well as a result of treatment with different primaquine regimens.

**Conclusions:**

A novel, simple, fast, and sensitive method to measure pre-patent period of malaria infection in rodents is described here. The sensitivity and accuracy of this new method is comparable to standard PCR and microscopy-based techniques, respectively.

## Background

Malaria infection is initiated when *Plasmodium* parasites enter the host through the bite of an infected *Anopheles* mosquito. The clinical manifestations of the disease occur during the intra-erythrocytic stage of replication, but prior to infecting red blood cells *Plasmodium* parasites go through an obligatory and asymptomatic phase of intrahepatic development.

Upon reaching the liver, sporozoites invade host hepatocytes inside which they develop into thousands of red blood cell-infectious merozoites [1]. Targeting this asymptomatic stage of infection is crucial for the success of anti-malarial interventions since clearance of the parasite at this stage completely abrogates the clinical manifestation and transmission of the disease. As such, the development of anti-malarial vaccine candidates and chemoprophylaxis approaches focused on the liver stage of infection is critical [2,3]. It is, however, challenging to evaluate the efficacy of anti-malarial strategies that target the development of *Plasmodium* parasites in the liver.

Rodent models are frequently used to study the impact of the different strategies *in vivo* and several methods have been developed to accurately measure *Plasmodium* liver infection (for a comprehensive review see [4]). Nevertheless, standard methods are invasive and terminal, as they require the collection of liver tissue to determine parasite burden. Among the usual methods are the microscopy-based techniques to analyse fixed livers upon immunofluorescence assay (IFA), using *Plasmodium*-specific antibodies, or live samples using transgenic parasites engineered to express fluorescent proteins [5] and quantitative reverse transcriptase PCR (qRT-PCR) to detect *Plasmodium*-specific targets, typically 18S ribosomal RNA, or trans-genes expressed by engineered parasites [6]. These assays are usually performed in the end phase of *Plasmodium* development in the liver (between 40 and 48 hr after infection with *Plasmodium berghei* or *Plasmodium yoelii* sporozoites), and prior to the egress of parasites into the blood. As such, they are inappropriate to evaluate the impact of the anti-malarial strategies on the final stage of the parasite maturation, egress from the liver and onset of the symptomatic blood infection [7].

The length of the pre-patent period, i.e. the time between sporozoite inoculation and the appearance of parasites in the blood, directly reflects the duration of the liver stage and the number of infective merozoites produced. However, determining the onset of blood parasitaemia through the analysis of blood smears by microscopy is time-consuming and not accurate, as it may take a few cycles of blood stage replication to achieve a detectable level of parasites in the blood. The molecular methods (PCR-based techniques) are more sensitive allowing not only to accurately measure parasite load but also to determine speciation. They are, however, expensive and labor intensive methods [8,9].

The recently developed chemiluminescent parasites have become valuable tools in experimental malaria research. The luciferase-expressing parasites can be used to assess parasite hepatic development through non-invasive methods and still allowing infection to proceed to the blood stage in the same animals where liver burden was previously assessed [10]. As such, it is possible, using these parasites to evaluate the effect of specific anti-malarial strategies that target the liver stage.

Here, a further use for these transgenic luciferase-expressing lines is proposed to accurately evaluate the pre-patent period of malaria infection. This new assay constitutes a simple, fast, non-invasive, and highly sensitive bioluminescent assay allowing a precise evaluation of the malaria pre-patent period as it allows the detection of blood parasites as early as 60 hr after sporozoite inoculation. This means that this method is able to detect the first generation of merozoites released from the liver, since that merozoite egress from hepatocytes starts 48 hr after infection with sporozoites [11].

## Methods

### Mice, parasites and reagents

Male C57BL/6 mice, aged six to eight weeks were purchased from Charles River and housed in the pathogen-free facilities of the Instituto de Medicina Molecular, Lisbon (IMM). The luciferase expressing *P. berghei* ANKA transgenic parasite lines (676m1cl1 line) was used in all the experiments. Sporozoites were obtained by disruption of the salivary glands of freshly dissected infected female *Anopheles stephensi* mosquitoes and collected in DMEM (Dulbecco’s Modified Eagle Medium from GIBCO). Mosquitoes were bred at the insectary facility of the Instituto de Medicina Molecular. Firely luciferase assay kit was purchased from Biotium, Hayward, USA and DNA extraction kit was purchased from QIAGEN.

### Ethics statement

All experimental procedures were performed according to EU recommendations for good practices and animal welfare and approved by the IMM Animal Care and Ethical Committee (AEC_2010_024_MM_RDT_General_IMM). Animals were monitored daily and every effort was made to minimize suffering. Upon completion of experiments, mice were euthanized via administration of CO_2_ followed by cervical dislocation.

### Infection and bioluminescence assay

*Plasmodium* liver stage infection was established by mosquito bite, through a 20 minutes bite of one infected *Anopheles* mosquito, or intravenously (IV) through retro-orbital injection of the designated quantity of sporozoites. At the selected time-points 1 μL of blood was collected from the tail vein into 50 μL of lysis buffer. Luminescence was determined by adding 50 μL of D-luciferin dissolved in firely luciferase assay buffer (according to the manufacturer’s instructions) to 30 μL of lysate and immediately measured using a multiplate reader (Tecan, Switzerland). Values of luciferase activity are expressed as relative luminescence units (RLU).

### DNA extraction and real-time PCR analysis

At the selected time-points, and concomitant with the sampling for bioluminescence assay, 1 μL of blood was collected from the tail vein into 200 μL of 1× PBS. DNA extraction was performed using the DNeasy Blood & Tissue Kit, according to the manufacturer’s instructions. Real-time PCR analysis to was performed using 2 μL of DNA and the iTaq Universal SYBR Green Supermix from Bio-Rad according to the manufacturer’s instructions. Expression levels of 18 s rRNA were normalized against the housekeeping gene seryl-tRNA synthetase (PbANKA_061540). Gene expression values were calculated based on the ΔΔCt method. Primer pairs used were: PbA 18S rRNA: 5′GGAGATTGGTTTTGACGTTTATGTG3′ and 5′GGAGATTGGTTTTGACGTTTATGTG3′; PBANKA_061540: 5′ATTGCTCAACCTTATCAAACTG3′ and 5′AGCCACATCTGAACAACCG3′.

### Microscopy analysis

Blood parasitaemia was monitored daily by light microscopy, starting 60 hr after infection. Air-dried thin blood smears were fixed in absolute methanol and stained with Giemsa. Percentage of infected red blood cells (iRBC) was assessed by microscopic examination of 15 fields of Giemsa-stained blood smears.

### Statistical analysis

Statistically significant differences between two different groups were analysed using the Mann–Whitney test. *P* < 0.05 were considered statistically significant. Significances are represented in the figures as follows *P < 0.05; **P < 0.01. All statistic tests were performed using Graph Prism 5.0 software.

## Results

### Bioluminescence assessment of blood parasite load correlates with parasitaemia measurements

**Figure 1:**
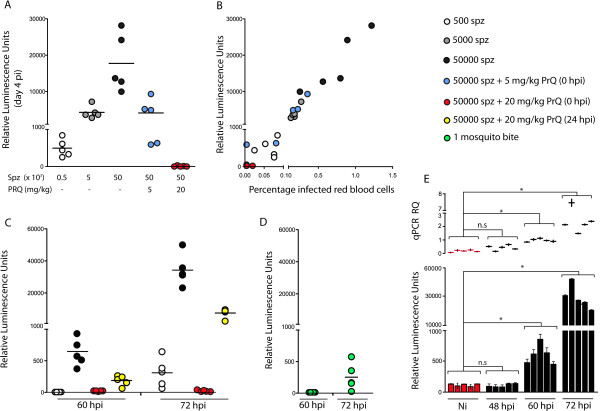
**Bioluminescence assessment of blood parasite load correlates with parasitaemia measurements and quantitatively detects circulating parasites at 60 hr after infection. A**: Bioluminescence measurements in the blood four days after infection with different numbers of luciferase-expressing *P. berghei* ANKA sporozoites (500, 5,000 or 50,000 injected IV), with or without primaquine treatment. **B**: Correlation of bioluminescence assessment with blood-stage parasitaemia determined by blood smear analysis at day 4 after infection with luciferase-expressing *P. berghei* ANKA sporozoites. **C**: Assessment of pre-patent period at 60 h and 72 h after infection with different number of sporozoites (500 or 50,000 injected IV) and with or without primaquine treatment **D**: Assessment of pre-patent period at 60 h and 72 h after infection transmitted by the bite of 1 infected mosquito. **E**: Comparison of bioluminescence measurement with qPCR analysis of parasite load at 48, 60 and 72 hr after infection with 50,000 sporozoites. Non-infected (Ni) blood samples are included as control. (A-D: Each dot represents the signal of one infected mouse. Bars represent the mean value. **E**: Each bar represents the bioluminescence signal of one infected mouse measured in technical triplicates. PCR analysis was performed in technical duplicates).

To determine if bioluminescence levels measured in total blood correlate with the sporozoite inoculum used to initiate infection, mice were infected with increasing numbers of luciferase-expressing *P. berghei* sporozoites (500, 5,000 and 50,000). Four days later luciferase activity was measured using 1 μL of tail-vein blood. The results clearly show that bioluminescence is detected 4 days after sporozoite infection and that its level is proportional to the number of sporozoites used to initiate infection (Figure 1A). In addition, the administration of primaquine (PRQ), clinically used to eliminate liver stage parasites, resulted in a significant reduction in the bioluminescence measurements in total blood in a dose-dependent manner, suggesting that the luminescence levels detected in the blood are directly proportional to the level of liver infection. Importantly, the bioluminescence measurements show a direct correlation with the level of parasitaemia quantified by blood smear monitoring (Figure 1B).

### Bioluminescence measurements detect circulating parasites 60 hr upon infection with 50,000 sporozoites

Having shown that bioluminescence measurements have a direct correlation with the number of circulating parasites, the next step was to establish the earliest time point after sporozoite infection, as well as the lowest level of parasites that this assay could detect. Infections were initiated by IV injection of 500 and 50,000 sporozoites and bioluminescence measurements from 1 μL of tail-vein blood were performed 60 and 72 hr after infection. The results show that circulating parasites can be quantified by this method as early as 60 hr when the infection is initiated with 50,000 sporozoites (Figure 1C). Importantly, the luminescence levels are proportionally lower upon administration of primaquine (PRQ) on the day of infection (estimated to reduce liver infection by 90%) or 24 hr after infection (estimated to reduced approximately 50% of liver parasite load). In addition, an infection initiated by the bite of one single infected mosquito could be detected 72 hr after the infectious bite (Figure 1D) in levels similarly to the inoculation IV of 500 sporozoites (Figure 1C).

### Sensitivity of the bioluminescence assay is similar to the PCR detection of blood parasite load

To further test the sensitivity of the assay, bioluminescence measurements were compared with real-time PCR analysis, both in samples of 1 μL of tail-vein blood of animals infected by IV injection of 50,000 sporozoites at 48, 60 and 72 hr after sporozoite infection. The results show that the sensitivity of both methods is similar, as in both cases the differences to non-infected (Ni) blood samples are only significant at 60 and 72 hr, but not at 48 hr after infection. The results of the analysis of technical triplicates are further indicators of assay robustness.

## Discussion and conclusions

The bioluminescent assay described here allows the fast and efficient quantitative monitoring of pre-patent period in a minimally invasive manner. This technique has clear benefits over the traditional, time-consuming, examination of blood smears to detect the presence of circulating parasites. It takes a few cycles of blood stage replication to produce a level of parasitaemia detectable by blood smear analysis; as such, the measurement of the pre-patent period by this method may be inaccurate and may include the impact of the anti-malarial strategy in the blood stage without providing a direct quantification of production and release of infective first-generation merozoites. Additionally, this assay presents similar sensitivity to the widely used PCR-based methods.

The requirement to use the chemiluminescent parasites further allows the combination with bioluminescence assays during liver stage infection to better discriminate the effects of distinct intervention strategies. The recent reports of luciferase-expressing parasites of different species, including *P. yoelii*[12] as well as *Plasmodium falciparum*[13], will allow the expansion of this protocol to these species. Most importantly, the description of liver and blood-humanized mice (until now still in separate hosts) [14,15] may soon allow the establishment of this assay to test different strategies against *P. falciparum* infection. As such, the use of different luciferase-expressing parasites may greatly accelerate and streamline the process of identifying and analysing future vaccine candidates as well as drug candidates against the pre-erythrocytic stages of malaria parasites, including those that may target the parasite at very late states or transition period between stages.

## Competing interests

The authors declare that they have no competing interests.

## Authors’ contributions

VZL and JSD performed the experiments. MMM supervised the study. VZL, JSD and MMM wrote the manuscript. All authors read and approved the manuscript.
